# Neurotransmitters in tumors: chemical cross-talk shaping tumor progression

**DOI:** 10.1186/s40364-025-00835-6

**Published:** 2025-10-14

**Authors:** Wenting Chen, Jie Yu, Shengfang Ge, Tifei Yuan, Xianting Ding, Biao Yan, Lin Ye, Yefei Wang, Renbing Jia

**Affiliations:** 1 Department of Ophthalmology, Ninth People’s Hospital, Shanghai JiaoTong University School of Medicine, Shanghai, 200025 P.R. China; 2 State Key Laboratory of Eye Health, Shanghai Key Laboratory of Orbital Diseases and Ocular Oncology, Shanghai, P.R. China; 3 Shanghai Mental Health Center, Shanghai Jiao Tong University School of Medicine, Shanghai, P.R. China; 4 School of Biomedical Engineering, Shanghai Jiao Tong University, Shanghai, P.R. China; 5 Eye Institute, Department of Ophthalmology, Eye & ENT Hospital, State Key Laboratory of Medical Neurobiology, Fudan University, Shanghai, P.R. China; 6 National Health Commission Key Laboratory of Myopia (Fudan University, Chinese Academy of Medical Sciences, Shanghai, P.R. China; 7 Shenzhen Eye Medical Center, Shenzhen Eye Hospital, Southern Medical University, Shanghai, P.R. China

**Keywords:** Tumor, Neurotransmitter, Cross-talk, Tumor microenvironment

## Abstract

The role of innervation in the pathogenesis of malignancies has been documented in many investigations. Recent studies have revealed that neurotransmitters act as mediators in nerve-stimulated cancer progression by directly influencing tumor cells and modulating the tumor microenvironment, including immune cells, angiogenesis, and surrounding stromal cells. Notably, psychological stress has been identified as a contributing factor to oncogenesis, primarily mediated by neurotransmitters. However, the complex interplay between neurotransmitters and tumor cells remains only partially understood. In this review, we explore newly identified mechanisms through which neurotransmitters (acetylcholine, glutamate, serotonin, dopamine, adrenaline, noradrenaline, γ-aminobutyric acid, neurotensin, and neuropeptide Y) regulate cancer initiation and progression, along with potential therapeutic strategies derived from these findings.

## Introduction

Cancer, a leading cause of premature death, presents not only a significant public health challenge but also an economic and societal burden in the 21st century [1]. While cancer treatment has seen remarkable progress, with many therapies becoming more personalized and effective, challenges remain. One major reason lies not in a lack of understanding, but in the complexity of cancer pathogenesis, which collectively hinder the effectiveness of many therapies, including immunotherapy. The pathogenesis of cancer is complex, involving genetic mutations, epigenetic abnormalities, environmental factors, and so on. Interestingly, a study on prostate cancer first observed increased nerve density in cancerous areas [2]. Subsequent studies confirmed that the innervation of sensory, sympathetic, and parasympathetic nerves can influence tumorigenesis, indicating the important role of nerves in tumor initiation, progression and metastasis [3, 4].

Neurotransmitters were first identified as chemical substances synthesized within neurons, stored in secretory vesicles at nerve terminals, and released through exocytosis as first messengers in response to neuronal firing. Upon release, they bind to receptors on post-synaptic neurons to regulate electrical activity and are subsequently inactivated by neuronal reuptake or enzymatic degradation [5]. These signaling molecules are widely distributed in the central and peripheral nervous systems, where they govern vital functions such as emotion, sensation, memory, and movement. Based on their structure and chemical properties, neurotransmitters are classified into several families, including cholinergic transmitters (e.g., acetylcholine), biogenic amines (e.g., dopamine, adrenaline, norepinephrine, and serotonin), amino acids (e.g., glutamate and γ-aminobutyric acid), and neuropeptides (e.g., neurotensin and neuropeptide Y). Emerging evidence suggests that tumors exploit neurotransmitter signaling pathways to establish a supportive microenvironment for growth and survival. For instance, GABA has been shown to promote colon cancer proliferation and migration [6]. Additionally, breast and lung cancer cells cocultured with neurons expressed classical neurotransmitter receptors, enabling their adaptation to the central nervous system and facilitating brain metastasis [7]. In medullary thyroid cancer, calcitonin gene-related peptide has been found to suppress immune cells within the tumor microenvironmen [8]. Despite these findings, the intricate relationship between neurotransmitters and tumor cells remains incompletely understood and warrants further investigation.

In this review, we highlight key neurotransmitters that serve as major modulators in various cancers, including acetylcholine, glutamate, serotonin, dopamine, adrenaline, noradrenaline, γ-aminobutyric acid (GABA), neurotensin, and neuropeptide Y. We summarize current knowledge on the roles of these neurotransmitters in tumorigenesis and explore emerging therapeutic strategies targeting neurotransmitters, their receptors, and associated signaling pathways.

## Pro-tumor function of acetylcholine

**Fig. 1:**
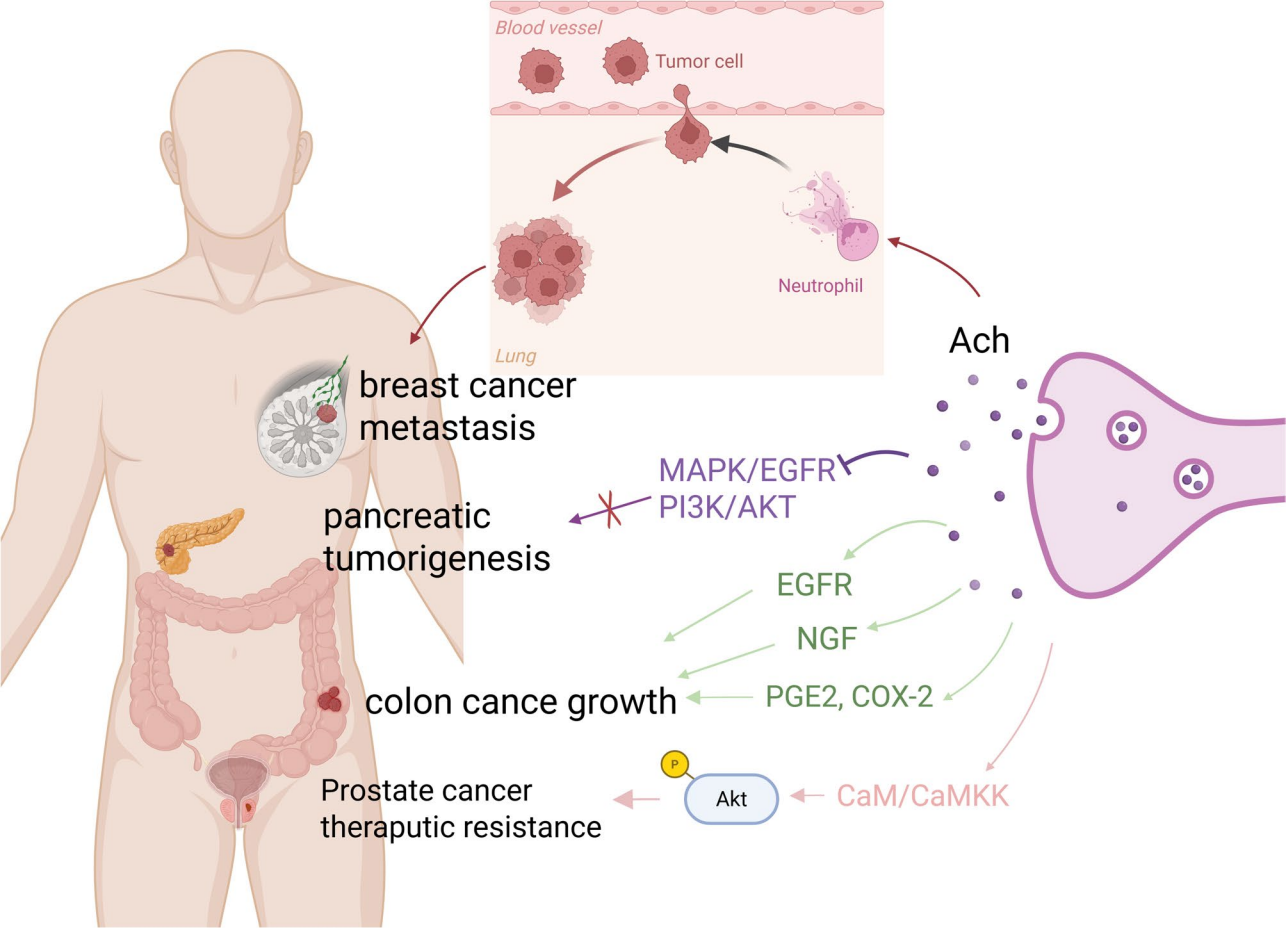
The role of acetylcholine in cancer progression. Acetylcholine (Ach) regulates tumor cell proliferation directly through receptors expressed on tumor cells. Upon activation, Ach receptors trigger intracellular calcium release and manipulate downstream signaling pathways to influence tumorigenesis. Arrows indicate promotion and horizontal lines indicate inhibition. Abbreviations: Ach, acetylcholine; MAPK, mitogen-activated protein kinase; EGFR, epidermal growth factor receptor; PI3K, phosphoinositide 3-kinase; AKT, protein kinase B; NGF, nerve growth factor; PGE2, prostaglandin E2; COX-2, cyclooxygenase-2; CaM, calmodulin; CaMKK, Ca2+/calmodulin-dependent protein kinase kinase

Acetylcholine (ACh), a neurotransmitter widely present in both the central and peripheral nervous system [9], facilitates communication between neurons and neuromuscular junctions, regulating essential functions such as muscle movement, heart rate, and respiration [10]. Besides, ACh also mediates immune cells functions [11]. Its receptors are classified into two major groups: muscarinic and nicotinic receptors, each comprising several subtypes [12]. These receptors are implicated in various diseases, including Alzheimer’s disease, schizophrenia, substance addiction, and tumorigenesis [13, 14]. Current research indicates that acetylcholine regulates tumorigenesis either by directly acting on ACh receptors expressed on tumor cells or by modulating immune cells to reshape the tumor microenvironment (TME) (Fig. 1).

Over the past three decades, researchers have discovered that acetylcholine stimulates tumorigenesis in various cancers [15, 16]. A key receptor mediating this effect is the cholinergic muscarinic type 3 receptor (CHRM3). Upon activation, CHRM3 promotes tumor cell proliferation by inducing intracellular calcium release [17]. Additionally, activated CHRM3 facilitates the progression of colon carcinoma by regulating PGE (2) and COX-2, two well-known gastrointestinal cancer promoters [18]. Acetylcholine also drives gastrointestinal carcinogenesis through the CHRM3-mediated ACh-NGF (nerve growth factor) axis [19]. In H508 human colon cancer cells, the interaction of cholinergic ligands with CHRM3 leads to EGFR transactivation, thereby stimulating cellular proliferation [20]. Furthermore, CHRM3 activation through autocrine cholinergic signaling promotes prostate cancer growth and confers therapeutic resistance via calmodulin/calmodulin-dependent protein kinase kinase (CaM/CaMKK)-mediated Akt phosphorylation [21]. Moreover, a study about GBM found that activation of metabotropic CHRM3 receptor by ACh reprograms GBM cells toward a more motile phenotype, enhancing tumour progression, whereas CHRM3 suppression reduces motility and prolongs survival [22].

Beyond CHRM3, recent studies on prostate tumorigenesis have highlighted cholinergic muscarinic type 1 receptor (CHRM1) as another key muscarinic receptor involved in tumor progression, with some evidence also implicating cholinergic muscarinic type 4 receptor (CHRM4). In prostate cancer (PCa), elevated expression levels of CHRM1 and CHRM3 have been observed [23]. Amplification or gain of their respective encoding genes correlates with worse prognosis and occurs more frequently in castration-resistant prostate cancer (CRPC) than in hormone-sensitive prostate cancer (HSPC), as PCa cells expressing CHRM1 and CHRM3 resist castration via the FAK (Focal Adhesion Kinase)-YAP (Yes1 Associated Transcriptional regulator) signaling axis [23]. Clinically, docetaxel is the most commonly used chemotherapy for CRPC and advanced prostate cancer. Research indicates that CHRM1 mediates docetaxel resistance, and its inhibition reverses resistance while suppressing tumorigenesis [24]. Additionally, CHRM4 has been reported to interact with NGF, thereby promoting neuroendocrine differentiation of prostate cancer cells, a process associated with tumor progression and therapy resistance [25].

Although substantial evidence supports the role of acetylcholine in promoting tumorigenesis, its effects appear to be site-specific. In contrast to its tumor-promoting effects described earlier, research has shown that CHRM1 activation can inhibit the MAPK/EGFR and PI3K/AKT pathways in pancreatic ductal adenocarcinoma (PDAC) cells, thereby suppressing tumorigenesis [26]. Furthermore, subdiaphragmatic vagotomy or CHRM1 knockout has been found to accelerate pancreatic tumorigenesis, partly through the expansion of the cancer stem cell (CSC) population, which can be mitigated by systemic administration of the muscarinic agonist bethanechol. Thus, targeting CHRM1 may represent a promising therapeutic strategy [26].

In addition to regulating tumor progression directly through acetylcholine receptors expressed on tumor cells, acetylcholine also influences the TME by modulating immune cells. Research has shown that brain activity can regulate immune responses in lymphoid organs through neurotransmitters, including acetylcholine [27]. Acetylcholine participates in immune regulation through various mechanisms. For instance, in the spleen, acetylcholine produced by memory phenotype T cells inhibits tumor necrosis factor-α (TNF-α) production by cytokine-producing macrophages [28]. This anti-inflammatory effect is mediated through the α7 nicotinic acetylcholine receptor (α7nAChR) and is initiated by vagus nerve stimulation, forming part of the inflammatory reflex arc that limits systemic inflammation [28]. In pancreatic ductal adenocarcinoma, perineural invasion-induced cholinergic signaling supports tumor growth by promoting an immune-suppressive microenvironment characterized by reduced CD8 + T-cell infiltration and a lower Th1/Th2 ratio [29]. Moreover, recent findings suggest that acetylcholine cooperates with psychological stress to remodel the TME [30]. Compared with patients with non-metastatic breast cancer, those with breast cancer metastasis to the lungs showed elevated acetylcholine expression [30]. This effect is attributed to chronic stress enhancing acetylcholine expression, which promotes neutrophil extracellular trap (NET) formation (NETosis) in the lungs. These NETotic neutrophils can capture cancer cells, thereby facilitating breast cancer lung metastasis.

Based on the mechanisms described above, targeting ACh receptors and their downstream signaling pathways may offer effective therapeutic strategies against tumors. Experimental studies in vitro and in mouse models have demonstrated the potential of such approaches [31]. Additionally, targeting ACh receptors could be incorporated into combinatorial strategies to overcome chemotherapeutic resistance [21].

## Pro-tumor function of glutamate

Glutamate is the most abundant excitatory neurotransmitter in the central nervous system (CNS) [32], playing a critical role in neural processes such as learning, memory formation, and neuronal maturation. It mediates excitatory neurotransmission through two receptor types: metabotropic glutamate receptors (mGluRs), which are G protein–coupled receptors, and ionotropic glutamate receptors (iGluRs), which are cation-permeable ligand-gated ion channels. The iGluRs are further classified into functional subtypes, including α-amino-3-hydroxy-5-methyl-4-isoxazolepropionic acid receptors (AMPARs), kainate receptors, N-methyl-D-aspartate receptors (NMDARs), and GluD receptors (also known as delta or δ receptors). Glutamate regulates long-term changes in neuronal excitability, synaptic structure and function, neuronal migration during development, and neuronal viability [32].

Although glutamate is essential for normal neural function, its signaling pathways can be exploited by tumor cells to promote oncogenesis. Accumulating evidence suggests that glutamate is secreted by various cancers, with its protumoral effects mediated through both mGluRs and iGluRs [33, 34]. Activation of mGluRs by glutamate regulates the proliferation of neural stem-progenitor cells, which are considered potential origins of malignant brain tumors. Specifically, mGlu (3) and mGlu (4) receptors control brain tumor cell proliferation, while mGlu (1) receptors are implicated in melanoma progression [35]. Besides, in brain metastases of human lung cancer, interactions with astrocytes induce mGluR1 expression via the Wnt-5a/PRICKLE1/REST pathway, leading to glutamate-dependent stabilization of EGFR and increased reliance on mGluR1 signaling, which represents a potential therapeutic vulnerability [36]. Furthermore, AMPA receptors facilitate electrochemical communication between neurons and gliomas via neuro-glioma synapses, leading to synchronized calcium transients in tumor-microtube-connected glioma networks that drive tumorigenesis [37].

Another glutamate receptor family, NMDARs, plays a critical role in the malignant progression of various cancer types. Unlike some other glutamate receptors, NMDARs in cancer cells are often activated through autocrine glutamate release mechanisms, such as VGLUT (vesicular glutamate transporter)-mediated secretion, intracellular glutamate from necrotic cells, or physiological levels of cytoplasmic glutamate [38]. Overexpression of NMDARs is observed in multiple cancers and correlates with poor prognosis [39–41]. For example, in pancreatic neuroendocrine tumors (PNETs), NMDAR expression is heightened at the invasive front. Under conditions mimicking interstitial fluid pressure, NMDAR activation, along with downstream MEK-MAPK and CaMK signaling, promotes invasiveness, suggesting that fluid flow-induced NMDAR activation may contribute to malignancy via autocrine glutamate signaling circuits [41]. Moreover, glutamate enhances invasiveness in PNET and pancreatic ductal adenocarcinoma through interactions with GKAP (guanylate kinase-associated protein), a scaffold protein of NMDAR, and its downstream effectors FMRP (fragile X mental retardation protein) and HSF1 (heat shock transcription factor 1) [42]. NMDAR signaling also facilitates neuronal pathways associated with invasive tumor growth, which are crucial for metastatic colonization of the brain. For breast-to-brain metastasis (B2BM), signaling is driven by pseudo-tripartite synapses formed between cancer cells and glutamatergic neurons, since the level of glutamate secreted by B2BM cells alone is insufficient to activate NMDAR signaling in both human and mouse models [39].

Given the role of glutamatergic synapses in driving tumor progression across various cancers, targeting glutamate receptors represents a promising therapeutic strategy. Experimental studies have demonstrated that glutamate antagonists can effectively limit tumor growth [43]. Specifically, simultaneous inhibition of NMDARs and AMPARs significantly impairs cancer cell proliferation and invasion, suggesting a novel therapeutic approach for cancer treatment.

## Pro-tumor function of serotonin (5-HT)

**Fig. 2:**
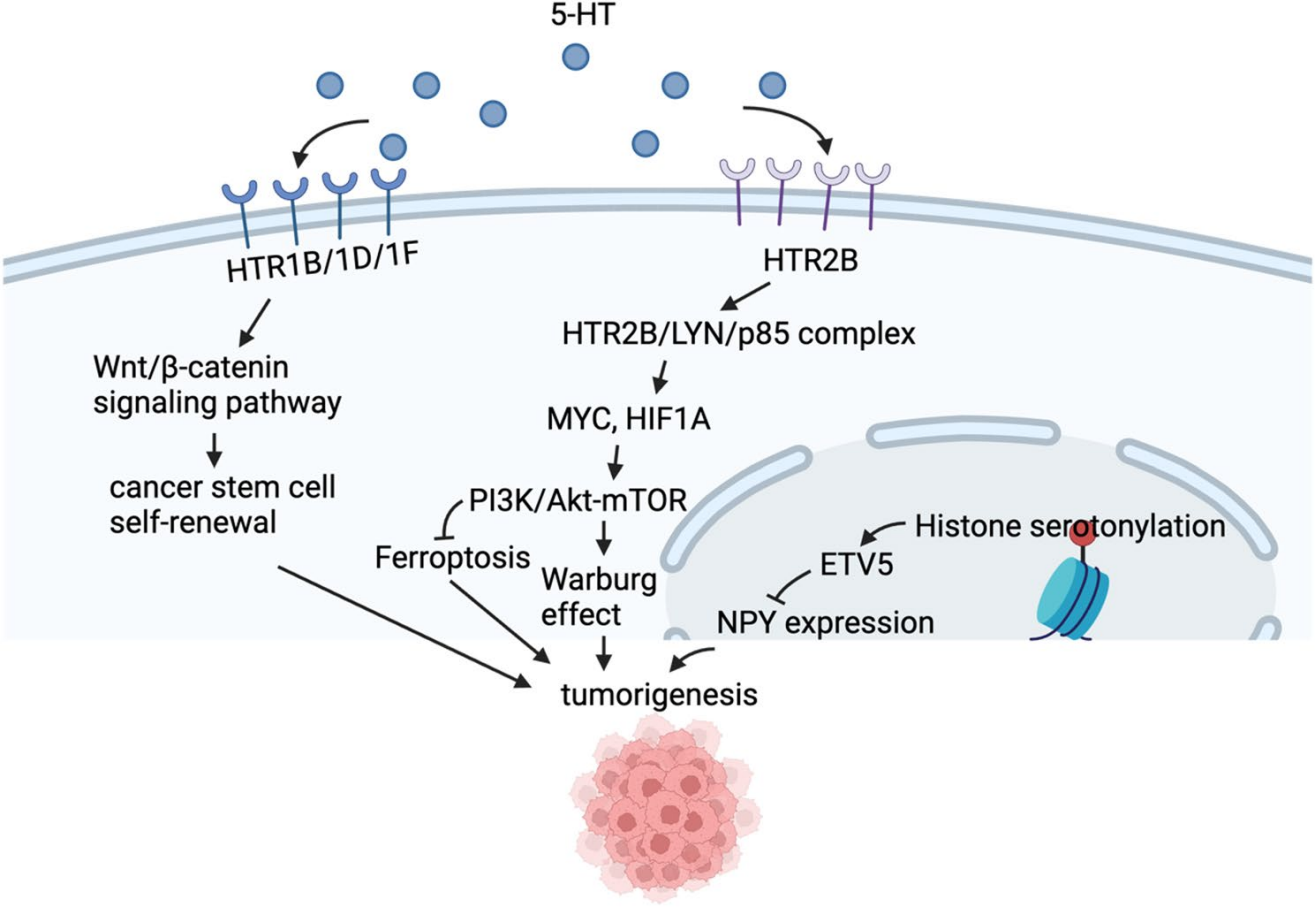
Serotonin promotes tumor progression. Serotonin (5-HT) regulates a broad range of physiological functions in the central and peripheral nervous systems. Its downstream signaling pathways can also be hijacked by tumors to stimulate tumorigenesis. Recently, it is reported that histone serotonylation can also promote tumor growth. Abbreviations: 5-HT, serotonin; HTR1B/1D/1F, 5-hydroxytryptamine receptor subtypes 1B, 1D, and 1 F; HTR2B, 5-hydroxytryptamine receptor 2B; LYN, tyrosine-protein kinase Lyn; p85, phosphoinositide 3-kinase regulatory subunit p85;MYC, myelocytomatosis oncogene; HIF1A, hypoxia-inducible factor 1-alpha; PI3K, phosphoinositide 3-kinase; Akt, protein kinase B; mTOR, mechanistic target of rapamycin; ETV5, ETS variant transcription factor 5; NPY, neuropeptide Y

Serotonin (5-HT) regulates a broad range of physiological functions in the central and peripheral nervous systems (CNS and PNS), including mood regulation, sleep, body temperature, inflammation, proliferation, regeneration, and tissue repair [44]. It acts as both a neurotransmitter and a paracrine messenger. In the enteric nervous system, 5-HT activates intrinsic and extrinsic primary afferent neurons to initiate peristaltic and secretory reflexes, respectively [45]. The biosynthesis of 5-HT begins with tryptophan hydroxylase (TPH), the first and rate-limiting enzyme [46]. TPH requires Fe²⁺ as a cofactor and uses O₂ and tetrahydrobiopterin (BH₄) as co-substrates to hydroxylate tryptophan, producing 5-hydroxytryptophan (5-HTP), which is subsequently decarboxylated by aromatic amino acid decarboxylase (AADC) to form 5-HT. Two TPH isoforms exist: TPH1, primarily found in peripheral glands such as the gut, and TPH2, restricted to the brain. 5-HT interacts with 15 receptor subtypes, grouped into seven families based on genetic and signaling characteristics, each structurally and functionally distinct [47]. Notably, 5-HT promotes tumor initiation and progression in several cancers, including gastrointestinal tumors, hepatocellular carcinoma, and pancreatic cancers (Fig. 2).

In colorectal cancer (CRC), microbiota metabolites induce tryptophan hydroxylase 2 (TPH2) expression in intestinal nerve cells, leading to 5-HT production. This 5-HT then interacts with overexpressed 5-HT receptors (HTR1B/1D/1F), activating the Wnt/β-catenin signaling pathway and promoting tumorigenesis through CSC self-renewal [48]. Additionally, 5-HT has been shown to reduce matrix metalloproteinase-12 (MMP-12) expression in tumor-infiltrating macrophages in colon tumors, resulting in decreased circulating angiostatin levels. Since angiostatin inhibits angiogenesis, reduced levels ultimately enhance tumor vascularity [44]. Elevated levels of serum and platelet-derived 5-HT have been observed in hepatocellular carcinoma (HCC) patients compared to healthy individuals [49]. Similarly, chemically induced HCC mouse models also exhibit increased serum 5-HT levels [50]. Another study demonstrated that starved HCC cells exhibited autophagy features that disappeared following 5-HT treatment [51]. 5-HT bypassed rapamycin, an mTOR inhibitor known to induce autophagy, through an mTOR-independent mechanism, activating downstream effectors such as p70S6K and 4E-BP1 [51]. In pancreatic ductal adenocarcinomas (PDACs), increased levels of 5-HT and enhanced expression of its receptor HTR2B facilitated tumor glycolysis under metabolic stress. Upregulated TPH1 and downregulated MAOA, enzymes responsible for 5-HT synthesis and degradation respectively, resulted in 5-HT accumulation. Elevated 5-HT levels promoted the formation of the HTR2B-LYN-p85 complex, leading to increased protein levels of MYC and HIF-1α, which ultimately activated PI3K-Akt-mTOR signaling and enhanced the Warburg effect [52]. Similarly, 5-HT has been implicated in tumor cell metabolism in human gastric adenocarcinoma. Its receptor HTR2B regulates lipid metabolism, inhibiting ferroptosis by interacting with Fyn to modulate p85 activity and trigger the PI3K/Akt/mTOR signaling pathway [53].

Notably, serotonyaltion has been discovered as a novel post-translational modification and function through receptor-independent ways. For example, intracellular 5-HT serves as a substrate for transglutaminase 2 (TG2)-mediated serotonylation of RhoA, which subsequently activates the RhoA/ROCK/YAP signaling pathway and promotes colorectal cancer progression [54]. A recent report identified a glyceraldehyde-3-phosphate dehydrogenase (GAPDH) serotonylation system that enhances the antitumor immune activity of CD8 + T cells. In these cells, serotonylation facilitated the cytoplasmic localization of GAPDH, inducing a metabolic shift toward increased glycolysis, which contributed to enhanced antitumor immunity [55]. In addition, 5-HT has been shown to chemically modify histones, with such modifications playing a critical role in gene transcription. For example, in serotonergic cells, the H3K4me3Q5ser modification is permissive, enhancing TFIID binding to H3K4me3, which is essential for cellular differentiation [56]. In ependymoma, elevated histone serotonylation promotes the expression of the transcription factor ETV5, which represses the expression of Neuropeptide Y (NPY), a neuropeptide with tumour-suppressive effects through synaptic remodelling. Inhibition of histone serotonylation suppresses tumour growth, partly by restoring NPY expression and attenuating tumour-associated neuronal hyperactivity [57]. In cancer-associated fibroblasts (CAFs), serotonylation of histone H3Q5 (H3Q5ser) triggers a switch toward an inflammatory-like CAF phenotype. This mechanism enhances CRC cell proliferation, invasiveness, and macrophage polarization, which can be reversed by knocking down the 5-HT transporter SLC22A3 or inhibiting TGM2 (thereby reducing H3Q5ser levels) [58]. A study about HCC pointed out that TGM2 promotes HCC progression via H3Q5ser-mediated activation of MYC target genes, and inhibition of its transglutaminase activity suppresses tumor growth and synergizes with sorafenib without notable toxicity—supporting TGM2 as a prognostic biomarker and therapeutic target [59]. Recently, a novel chemical probe for monoaminylation analysis was developed. Using this probe, it was confirmed that histone serotonylation and dopaminylation are highly enriched in tumor tissues overexpressing transglutaminase 2 (TGM2) and regulate the three-dimensional architecture of cellular chromatin [60].

The studies mentioned above provide insights into potential therapeutic strategies targeting the 5-HT pathway. For instance, 5-HT signaling can be disrupted by blocking SERT to inhibit the 5-HT–SERT–YAP axis, depleting 5-HT cargo, or using a TPH1 inhibitor to suppress 5-HT production [54]. Notably, peripheral serotonin has been shown to modulate the tumor immune microenvironment by promoting immunosuppressive macrophage polarization and impairing CD8⁺ T cell activity [44]. Consequently, inhibition of serotonin signaling not only suppresses tumor growth but also enhances the efficacy of immune checkpoint blockade such as PD-1 inhibitors [61]. Therefore, combining PD-1 blockade with serotonin-targeted therapies may yield synergistic antitumor effects [44, 54, 61]. Given that serotonylation plays distinct roles in tumor cells and immune cells, and 5-HT is widely present in both the central and peripheral nervous systems, therapeutic approaches targeting 5-HT and serotonylation must be highly specific to maximize benefit and minimize off-target effects.

## Pro-tumor function of dopamine

**Fig. 3:**
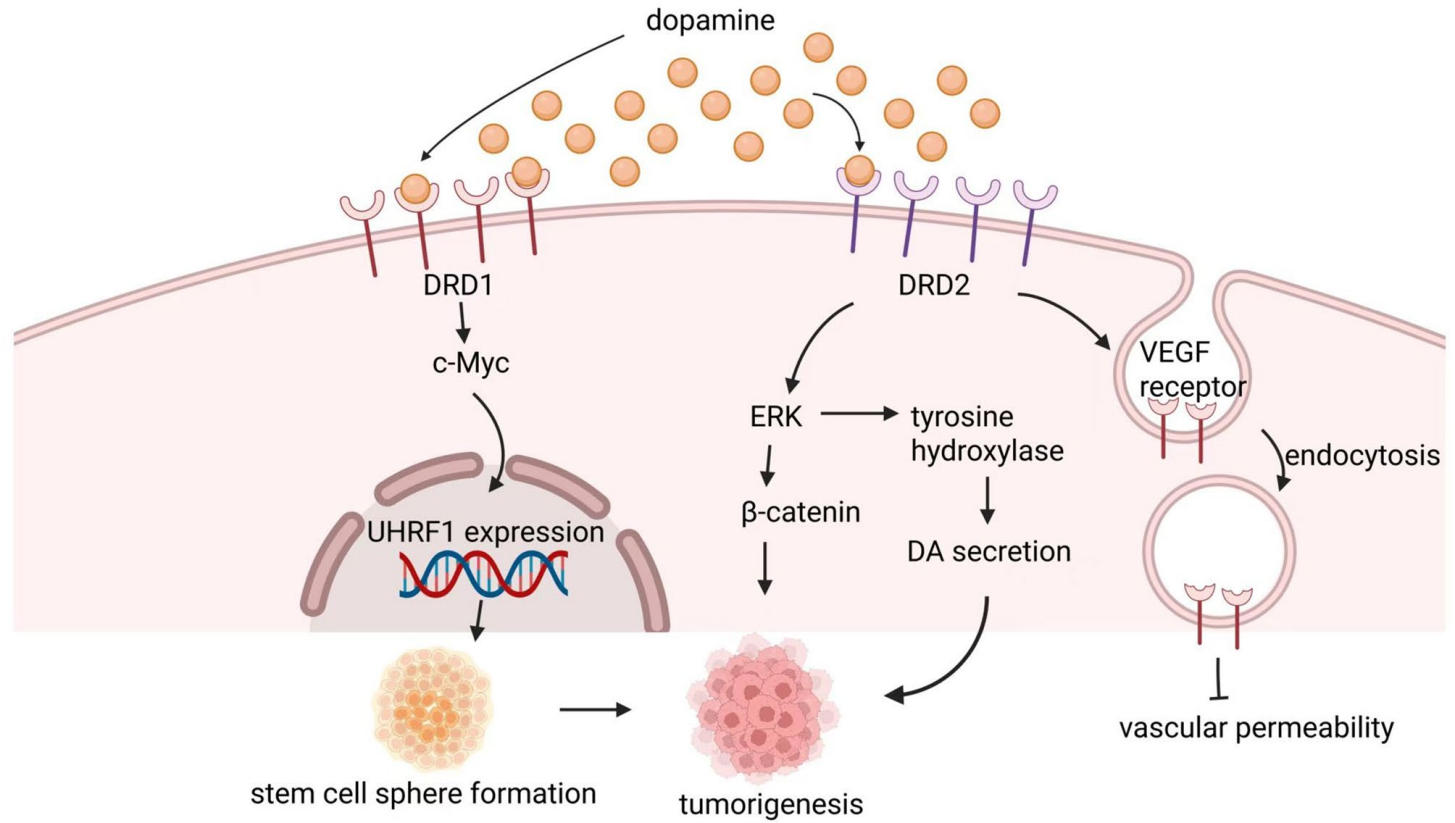
Dopamine plays an important role in oncogenesis. It manipulates gene expression, regulates signaling pathways and influences angiogenesis. Abbreviations: DRD1, dopamine receptor D1; DRD2, dopamine receptor D2; c-Myc, cellular myelocytomatosis viral oncogene homolog; UHRF1, ubiquitin-like with PHD and ring finger domains 1; ERK, extracellular signal-regulated kinase; β-catenin, beta-catenin; DA, dopamine; VEGF, vascular endothelial growth factor

Dopamine (DA) is a major catecholamine neurotransmitter that is widely distributed in both the CNS and PNS. In the brain, DA regulates various functions, including cognition, emotion, locomotor activity, and positive reinforcement. In the periphery, it influences the functions of several organs, including the cardiovascular system, kidneys, and gastrointestinal tract [62]. Initially, scientists observed two DA receptors [63, 64], one closely coupled to adenylyl cyclase (AC) and was named D1, the other didn’t and was named D2. As gene cloning procedures was introduced, more DA receptors were characterized, which were called D_3_ [65], D_4_ [66], and D_5_/D_1b_ [67]. D_5_/D_1b_ shares high homology with D1 in structure and pharmacology, while D3 and D4 are similar to D2 [65]. Although dopamine plays a critical role in regulating various physiological functions, its involvement in cancer progression remains unclear. Some studies have suggested that it plays an important role in oncogenesis through multiple pathways (Fig. 3).

First, it has been demonstrated that dopamine can modulate angiogenesis in tumors. Activation of D2 receptors leads to the endocytosis of VEGF receptor 2, a critical receptor for promoting angiogenesis, thereby inhibiting the vascular permeability factor/vascular endothelial growth factor (VPF/VEGF) pathway and its subsequent signaling events, ultimately reducing vascular permeability [68]. In addition to its inhibitory effects, dopamine can also promote tumor vessel normalization [69]. Given that inadequate antitumor drug delivery is partially attributed to abnormal tumor blood vessels, dopamine may enhance the chemotherapeutic efficacy of doxorubicin by improving tumor vessel normalization.

Dopaminergic signaling is also linked to CSCs. A study demonstrated that thioridazine, an antitumor drug, selectively targeted CSCs responsible for initiating leukemic disease and antagonized dopamine receptors expressed on these cells, suggesting that dopamine receptors (DR) may serve as biomarkers for various tumors [70]. In glioblastoma, inhibition or knockdown of the D1 dopamine receptor (DRD1) suppressed tumor growth, as well as glioma stem cell sphere formation and invasion. Mechanical study found that DRD1 regulated expression of UHRF1 gene by modulating c-Myc entry into the nucleus [71].

More importantly, numerous studies have demonstrated that psychological stress can drive tumorigenesis, with dopamine regulation identified as a key underlying mechanism. For instance, it was observed that chronic stress upregulated DA and D2 dopamine receptor (DRD2) levels in glioblastoma (GBM) tumor tissues. This upregulation led to the activation of β-catenin and an increase in tyrosine hydroxylase (TH) levels through the DRD2/ERK/β-catenin and DRD2/ERK/TH signaling axes, respectively. TH then stimulated DA secretion, forming an autocrine positive feedback loop [72]. Notably, another study on ovarian cancer found that chronic stress significantly decreased dopamine levels and increased tumor growth. This effect could be reversed by dopamine treatment, which blocked stress-induced angiogenesis and stimulated apoptosis [73]. Additionally, in the nuclei of stress-stimulated tumor cells, high expression of DRD2 and hypoxia-inducible factor-1α (HIF-1α) was observed. The competitive binding of DRD2 and HIF-1α to von Hippel-Lindau (VHL) resulted in reduced HIF-1α degradation and promoted epithelial-mesenchymal transition in tumor cells [74]. Moreover, a study on HCC found that local dopamine, a key mediator of tumor proliferation and metastasis, increased while dopamine metabolism became unbalanced, characterized by upregulation of dopa decarboxylase (DDC) and downregulation of monoamine oxidase A (MAOA) [75].

Thus, dopamine and its receptors may serve as biomarkers for predicting the prognosis of specific cancers, such as glioblastoma and HCC, where dysregulated dopamine signaling has been implicated [72, 75]. The study about glioblastoma has confirmed that DR inhibitors can suppress oncogenesis, offering potential therapeutic strategies [72]. Moreover, patients undergoing chemotherapy may experience improved treatment outcomes by adding dopamine to promote vessel normalization [69]. However, dopamine exerts opposing effects on tumor cells in different cancers [74, 75], necessitating further research to elucidate the underlying mechanisms and guide optimal therapeutic management.

## Pro-tumor function of adrenalin and noradrenaline

**Fig. 4:**
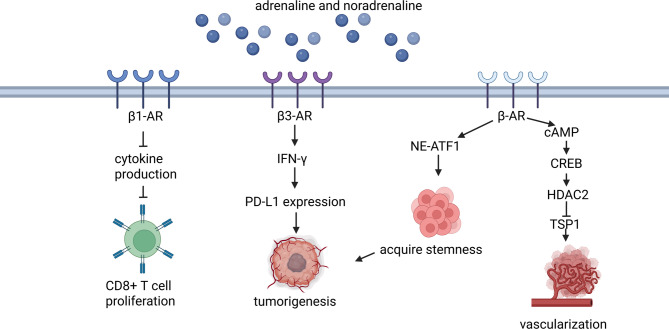
Signaling pathways of adrenalin and noradrenalin in tumorigenesis. Adrenalin and noradrenaline are crucial neurotransmitters and hormones. They exert stimulatory effects on cancer initiation and progression via inhibiting CD8 + T cells, directly regulating tumor cells and promoting vasculation. Abbrevations: β-AR, beta-adrenergic receptor; cAMP, cyclic adenosine monophosphate; IFN-γ, interferon-gamma; PD-L1, programmed death-ligand 1; NE-ATF1, nuclear-encoded activating transpcrition factor 1; CREB, cyclic AMP-responsive element-binding protein; HDAC2, histone deacetylase 2; TSP1, thrombospondin-1

Adrenaline (epinephrine) and noradrenaline (norepinephrine) are crucial neurotransmitters and hormones that regulate cardiovascular function, energy metabolism, stress response, and other physiological processes [76]. The biological effects of adrenaline and noradrenaline are mediated through various adrenergic receptor subtypes, which are G protein-coupled receptors [77]. Based on sequence homology, drug specificity, and mechanisms of signal transduction, these receptors are classified into two groups: α-adrenergic and β-adrenergic receptors, each with multiple subtypes [78]. Numerous studies have demonstrated that both adrenaline and noradrenaline exert stimulatory effects on cancer development and progression through these receptors [79–81] (Fig. 4).

Numerous studies have highlighted the critical role of β-adrenergic receptors (β-ARs) in tumorigenesis. First, β-ARs directly influence tumor growth and progression. An analysis of patient samples revealed that the density of sympathetic and parasympathetic nerve fibers in both tumor and surrounding normal tissue contributes to prostate cancer progression. This finding was further validated in a mouse model of prostate cancer, where depletion of β2- and β3-adrenergic receptors via sympathectomy or genetic deletion suppressed tumor growth, cholinergic-induced metastasis, and improved survival in mice [82]. Another study reached a similar conclusion, showing that norepinephrine (NE) accelerated high-grade serous ovarian cancer progression through β-AR signaling [83]. Additionally, biobehavioral risk factors such as social isolation and chronic restraint stress were found to be closely associated with tumor growth, with β-adrenergic signaling involved in these effects [84]. Research on various cancers suggests that these negative factors are linked to increased adrenergic signaling and elevated levels of NE, as well as the activation of β-adrenergic-related transcription factors, including NF-κB, AP-1, CREB, and STAT3 [80, 84]. Notably, this stress-induced effect could be reversed by β2-AR antagonists, whereas β1-AR antagonists had no such effect. Interestingly, eustress appears to have the opposite impact; evidence suggests that a peripheral neuroendocrine-immune pathway activated by eustress alleviates tumor immunosuppression, overcomes immunotherapy resistance, and enhances anti-tumor immunity through β-AR signaling, leading to reduced CCL2 expression [85]. Moreover, β-AR signaling can be exploited by cancer cells across diverse tumor types to acquire stemness through activation of the norepinephrine–ATF1 pathway [85]. Specifically, ATF1 cooperates with nuclear pluripotency factors and mitochondrial biogenesis regulators to enhance cancer stemness [86].

Most importantly, more and more evidence supports that beta-adrenergic signaling serves as a key role in manipulating immune cells in tumor microenvironment to drive tumor progression and metastasis, and some mechanisms were discovered. For example, stress responses induce CD8^+^T cell exhaustion via β_1_-adrenergic receptor expressed in CD8^+^T cell. β_1_-adrenergic signaling suppresses cytokine production and proliferation of CD8^+^T cell. And inhibition of β_1_-adrenergic signaling combined with immune checkpoint blockade (ICB) improves effector functions in melanoma [87]. Except for CD8^+^T cells, M2 macrophages were also reported influenced by β-adrenergic signaling. In breast cancer, β-adrenergic activation increased the infiltration of CD11b⁺F4/80⁺ tumor-associated macrophages with indications of M2 differentiation, thereby inducing a prometastatic gene expression signature and ultimately promoting cancer metastasis [88]. It was also pointed that myeloid-derived suppressor cells (MDSCs) inhibited antitumor immunity, in which β2-AR acted as an essential regulator [89]. On the one hand β2-AR stress pathway alters metabolism of MDSCs, like decreasing glycolysis and increasing fatty acid oxidation and PGE2, an immunosuppressive mediator [89]. On the other hand, it modulates expression of immunosuppressive molecules such as arginase-I and PD-L1 depending on STAT3 phosphorylation and increases MDSC survival through Fas-FasL interactions [90]. Another study found that regulatory mucosa-associated invariant T cells, presenting high immunosuppressive potential and promoted by β1 adrenergic receptor signaling, contributed to hepatocellular carcinoma progression and associated with poor clinical outcomes [91]. β3-adrenergic signaling impairs anti-tumor immunity by sustaining IFN-γ-dependent PD-L1 expression on tumor-infiltrating lymphocytes [92]. β-AR signaling can impair CD40-mediated anti-tumor immunity by rewiring downstream signaling in dendritic cells (DCs), including inhibiting IκBα phosphorylation and enhancing pCREB levels, thereby reducing the efficacy of αCD40 therapy in immunologically cold tumors [93]. On the contrary to the malignant effects mentioned above, it is found that exercise-induced adrenaline increases in mice reduced tumor incidence and growth by mobilizing IL-6 sensitive NK cells via β-adrenergic receptor [94]. Except for β_1_-adrenergic receptors, it is observed in multiple immunocompetent tumor models including immune checkpoint-blockade-based immunotherapy-resistant tumor that α2-adrenergic receptors (α2-AR) also have strong anti-tumor activity by increasing infiltrating T lymphocytes, reducing MDSC and upregulating innate and adaptive immune response pathways in macrophages and T cells [95].

β-AR also modulate the tumor microenvironment to promote tumor progression by stimulating vascularization. In vitro studies and mouse xenografts have shown that β-AR signaling activates cAMP response element-binding protein (CREB), which targets histone deacetylase-2 (HDAC2), an epigenetic regulator [96]. HDAC2 then represses thrombospondin-1 (TSP1), a potent inhibitor of vascularization, ultimately promoting angiogenesis [96]. β2-AR signaling enhances vascularization via receptors expressed on endothelial cells, altering their metabolism, such as suppressing oxidative phosphorylation [97]. Additionally, the cAMP-PKA signaling pathway activated by chronic stress through the β2-AR represents another potential mechanism [98].

Above studies offer promising therapeutic strategies against adrenalin and noradrenaline with significant potential. Evidence show that in breast cancer, β-blocker administration has been shown to effectively suppress tumorigenesis and improve survival outcomes, as demonstrated in clinical trials [99, 100].

## Pro-tumor function of γ-aminobutyric acid

γ-Aminobutyric acid (GABA), synthesized from glutamate by glutamate decarboxylase (GAD), is a major inhibitory neurotransmitter in the CNS [101]. The concentration of extracellular GABA is regulated by GABA transporters (GATs), which include four subtypes: GAT1, GAT2, GAT3, and GAT4 [102]. GABA binds to three receptor types: GABAA receptors (GABAAR) and GABAB receptors (GABABR), both ionotropic chloride channels, and GABAC receptors (GABACR), which are part of the G protein-coupled receptor (GPCR) family [103]. GABAAR are combined of different subunits, which include α (1–6), β (1–3), γ (1–3), δ, ε, θ, π, and ρ [104]. GABA is found in the CNS, PNS, and various peripheral tissues, including the gastrointestinal tract and liver [105, 106]. In addition to its normal functions—such as regulating neuronal activity, maintaining balance in the nervous system [107] and mediating inflammation [108], increasing evidence suggests that GABA is closely linked to cancer development. For instance, GABA synthesized and secreted by B cells and plasma cells promotes the differentiation of monocytes into anti-inflammatory macrophages that secrete IL-10 and suppress antitumor immunity [109].

GABA has been shown to be associated with poor prognosis in various tumors, promoting tumor progression either through its receptors expressed on tumor cells or by modulating the TME. Overexpression of GPT2 has been reported to promote breast cancer metastasis by increasing GABA levels and activating GABAAR. This activation triggers the PKC-CREB pathway via intracellular Ca²⁺ influx through calcium channels. The activated transcription factor CREB subsequently upregulates metastasis-related gene expression such as PODXL, MMP3, and MMP9, accelerating cancer metastasis [110]. In melanoma, GABA synthesized by melanocytes acts on GABAAR in keratinocytes. This electrical activity within the skin microenvironment modulates the capacity of melanoma driver oncogenes, such as *BRAF*^*V600E*^ to initiate melanoma [111]. Additionally, certain subunits of GABAAR, such as the π subunit (GABRP), can influence tumor growth [112]. In pancreatic ductal adenocarcinoma (PDAC) cells, GABRP overexpression has been observed. For GABRP-expressing PDAC cells, adding GABA to the culture medium enhances tumorigenesis by activating the mitogen-activated protein kinase/extracellular signal-regulated kinase (MAPK/ERK) pathway. Conversely, knockdown of endogenous GABRP expression or the use of a GABAA receptor inhibitor yields the opposite effect [112]. Another study demonstrated that GABRP activates nuclear factor κB (NF-κB) signaling via Ca²⁺ influx, leading to the expression of CXCL5 and CCL20 and ultimately inducing macrophage infiltration in PDAC [113]. Furthermore, it has been shown that GABAAR α1 expressed in LGR5⁺ intestinal stem cells (ISCs) contributes to chemoradiotherapy-induced toxicity in tumor treatment [114].

In addition to GABAAR, GABABR is also closely associated with tumorigenesis [116]. A study demonstrated that GABABR activation by GABA inhibits GSK-3β activity, leading to enhanced β-catenin signaling. This promotes tumor cell proliferation while suppressing CD8⁺ T-cell intratumoral infiltration [114]. Interestingly, GABABR1, the primary subunit of GABABR, is more frequently expressed in normal tissues and colorectal cancer patients with better prognoses. The study confirmed that GABABR1 suppresses tumor progression by inhibiting epithelial-mesenchymal transition (EMT) and the Hippo/YAP1 pathway [115]. Moreover, research on cholangiocarcinoma suggested that all three GABA receptors (GABAAR, GABABR, and GABACR) can inhibit tumor proliferation [117]. In vitro experiments demonstrated that GABA exerts a time- and dose-dependent inhibitory effect, which could be reversed by blocking GABA receptors. In vivo experiments further revealed that GABA reduces tumor size and proliferation [117].

## Pro-tumor function of neurotensin and neuropeptide Y

Neuropeptides are a distinct family of neurotransmitters, differing significantly from classical transmitters. They encompass more than fifty subtypes [118], ncluding substance P, neuropeptide Y, opioids, vasoactive intestinal polypeptide (VIP), bombesin, gastrin, and neurotensin. Unlike classical neurotransmitters, neuropeptides are cleared slowly from release sites and can diffuse over relatively long distances [119, 120]. Functioning as first messengers, neuropeptides mediate intercellular communication through multiple modes, consistent with the four fundamental forms of signaling in multicellular organisms: autocrine signaling, paracrine signaling, signaling through gap junctions, and endocrine signaling [121]. Recent studies have identified neuropeptides as promoters of tumorigenesis [122], closely linking them to homeostasis, immune function, and tumor metabolism [123].

Here, we focus on a few representative neuropeptides—such as substance P (SP) and neuropeptide Y (NPY)—because their tumor-promoting roles and mechanisms have been better characterized in recent studies, whereas others (e.g., bombesin, gastrin) have been less extensively studied in the cancer context and are thus only briefly mentioned.For example, the neuropeptide substance P (SP), one of the neuropeptide subtypes, can promote progression, invasion, and metastasis in breast cancer as well as in other malignancies, leading to reduced survival outcomes [124]. SP exerts its effects mainly by binding to the neurokinin-1 receptor (NK1R), the protein product of the human tachykinin receptor 1 (TACR1) gene (also referred to as the TACR1 receptor). In one study [124], SP activation of TACR1/NK1R on tumor cells led to cell death in TACR1-high cancers, with the dying cells releasing single-stranded RNAs (ssRNAs) that acted on neighboring tumoral Toll-like receptor 7 (TLR7) to trigger a pro-metastatic gene expression program. In another report [3], SP produced by intratumoral nerve fibers activated NK1R/TACR1 expressed on tumor cells to stimulate tumorigenesis, highlighting that SP–TACR1/NK1R signaling can have distinct, context-dependent effects on cancer biology. Neuropeptide Y (NPY), another subtype of neuropeptides, is reported to correlate with oncogenesis in HCC [125]. It is secreted by adjacent liver cells and act on NPY5 receptors expressed on tumor cells [125]. In prostate cancer, patients under psychologic depression presented higher NPY, which promoted myeloid cell infiltration and IL-6 release via STAT3 signaling pathway, ultimately facilitating tumor progression [126].

Building upon the mechanisms discovered, potential therapeutic strategies have been proposed. Targeting neuropeptides or their receptors shows promise in antitumor treatments. For instance, in breast cancer, aprepitant, an anti-nausea drug that targets the TACR1, was found to suppress tumor growth [124]. Similarly, in prostate cancer, inhibiting NPY may serve as a promising strategy for patients experiencing psychological depression [126].

## Neurotransmitter signaling in cancer metastasis

Metastasis, the leading cause of cancer mortality, is increasingly shaped by neurotransmitter signaling. For example, SP from breast cancer cells promotes metastasis via TACR1^high^ cell death and TLR7 activation, while the TACR1 antagonist aprepitant blocks this effect [124]. Likewise, ACh drives intrahepatic cholangiocarcinoma metastasis through CHRNA5 (alpha 5 nicotine acetylcholine receptor subunits)–CAMKII (Ca/calmodulin-dependent protein kinases) signaling, and CAMKII inhibition with KN93 markedly suppresses migration [127]. These findings suggest neurotransmitter pathways as promising therapeutic targets against metastasis.

## Neurotransmiters in neuroendocrine tumors

Neuroendocrine tumors (NETs) exemplify cancers in which neurotransmitters are central to both pathology and clinical features [128]. Recent studies show that neurotransmitters critically shape NET biology. For instance, calcitonin gene–related peptide (CGRP) promotes immunosuppression in medullary thyroid cancer (MTC) through cAMP (cyclic adenosine monophosphate)–KLF2 (Kruppel Like Factor 2) signaling [8]. Likewise, 5-HT produced by neuroendocrine prostate cancer (NEPC) induces neutrophil extracellular trap formation via histone 3 modification, driving liver metastasis [129]. These observations underscore the need for further investigation into neurotransmitter–NET interactions to inform therapeutic strategies.

## Conclusion

**Fig. 5:**
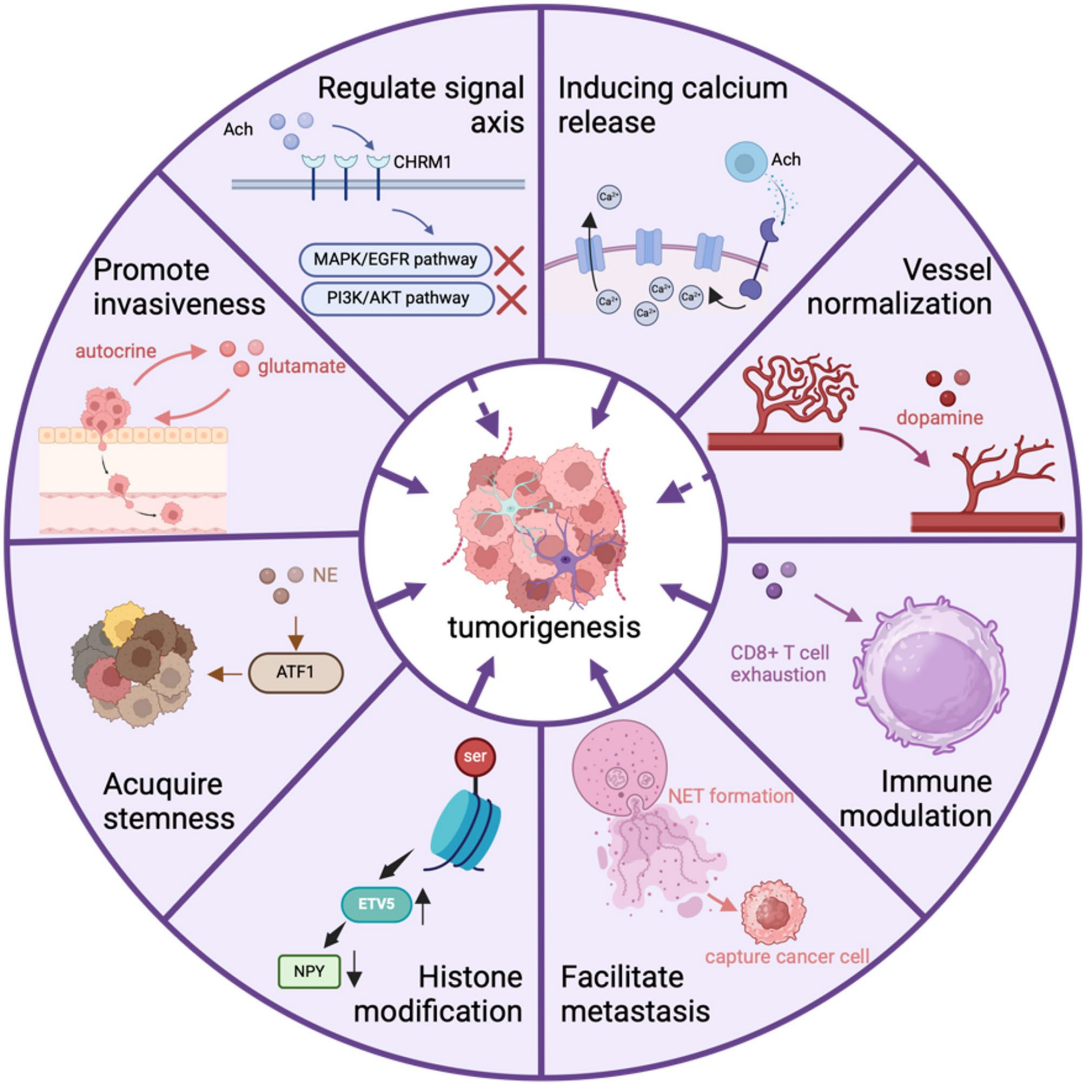
Mechanisms by which neurotransmitters promote carcinogenesis and development. Potential mechanisms include epigenetic modification, activation of carcinogenic signaling pathways, immune modulation, vessel normalization and induction of invasion and metastasis. Abbrevations: Ach, acetylcholine; NET, norepinephrine transporter; NE, norepinephrine; ATF1, activating transcription factor 1; CHRM1, cholinergic receptor muscarinic 1

The relationship between neurotransmitters and tumors has been validated by multiple studies. Neurotransmitters are secreted not only by neurons but also by intestinal epithelial cells, immune cells, neighboring stromal cells, and tumor cells. While neurotransmitters predominantly drive tumor progression through various pathways, they occasionally exhibit suppressive effects. They regulate tumor metabolism and gene expression, ultimately influencing tumorigenesis (Table 1, Fig. 5). Additionally, neurotransmitters modulate the TME by acting on receptors expressed by immune cells (Table 2). Furthermore, neurotransmitters mediate the impact of psychological stress on tumors, which aligns with their role as key regulators of emotional states. Except their tumor-stimulating effects mentions above, we want highlight that some studies also pointed out that neurotransmitter have anti-tumor impact [95, 117], such as restoring immune activity by reducing tumor immunosuppression [85], recruiting IL-6–sensitive NK cells [94]. Further research into how neurotransmitters influence tumor initiation, progression, and metastasis will significantly enhance our understanding of tumor aggressiveness. This knowledge holds potential for improving tumor management by enabling the prediction of metastasis and prognosis [130]. It also facilitates the development of therapeutic strategies targeting neurotransmitters, their receptors, downstream signaling, and associated metabolic pathways, some of which have already demonstrated efficacy.

**Table 1:** Neurotransmitters and receptors and their effects on tumor cells

	Receptors	Effect on tumor cells	Supporting details	Reference	
Acetylcholine (Ach)	Muscarinic and nicotinic receptors	Promote tumor cell proliferation and metastasis, mediate chemotherapy resistance	Stimulate tumorigenesis via intracellular calcium release; regulating colon cancer promotors PGE (2) and COX-2; regulating ACh-NGF axis to drive gastrointestinal caricinogenesis; activating EGFR transactivation to stimulate cellular proliferation in colon cancer; interacting with nerve growth factor in prostate cancer cells to promote neuroendocrine differentiation.		[17–20, 25]
		Suppress tumorigenesis	Inhibit MAPK/EGFR and PI3K/AKT pathway in pancreatic ductal adenocarcinoma		[26]
Glutamate	mGluRs and iGluRs (including AMPA, NMDA, GluD)	Influence the cells of origin of brain tumors	Regulate the proliferation of neural stem-progenitor cells		[35]
		Promote tumorigenesis	Facilitate electrochemical communication between neurons and gliomas, leading to calcium transients		[37]
		Promote invasiveness	Activate downstream MEK-MAPK and CaMK signaling pathways; interact with GKAP in PNET and PDAC		[41, 42]
Serotonin	HTR	Promote tumorigenesis	Drive cancer stem cell self-renewal in colorectal cancer		[48]
		Inhibit tumor cell autophagy	Even when mTOR is inhibited by rapamycin (an exogenous mTOR inhibitor), serotonin activates downstream signals (p70S6K, 4E-BP1) via an mTOR-independent pathway		[51]
		Facilitate tumor glycolysis in PDAC	Form HTR2B-LYN-p85 complex and enhance Warburg effect		[52]
		Inhibit ferroptosis in gastric adenocaricinoma	Modulate p85 activity and trigger PI3K/Akt/mTOR signaling pathway		[53]
Dopamine	DRD1	Tumor initiation	Target cancer stem cell (CSC) to initiate leukemic disease, stimulate stem cell formation and invasion through regulating *UHRF1* gene in glioblastoma		[70, 71]
	DRD2	Promote tumor proliferation and metastasis in glioblastoma	Reduce HIF-1α degradation and promote epithelial-mesenchymal transition		[74]
Adrenalin and noradrenaline	β-adrenergic receptors	Promote various cancer progression	Relate with transcription factors such as NF-κB, AP-1, CREB, and STAT3		[80, 84]
		Overcome immunotherapy resistance and enhance anti-tumor immunity	Eustress activate peripheral neuroendocrine-immune pathway, ultimately reduce CCL2 expression		[85]
		Acquire cancer cell stemness in diverse tumor types	Activate noradrenaline-ATF1 pathway		[86]
γ-Aminobutyric acid (GABA)	GABAAR,	Promote breast tumor progression	Trigger PKC-CREB pathway and upregulate metastasis-related gene expression, such as *PODXL*,* MMP3*		[110]
		Melanoma initiation	Trigger electrical activity to regulate the ability of oncogenes, such as *BRAF*^*V600E*^		[111]
	GABAAR, GABABR and GABACR	Suppress cholangiocaricinoma	Inhibit epithelial-mesenchymal transition (EMT) and the Hippo/YAP1 pathway		[115]
Neuropeptide substance P	tumoral tachykinin receptors (TACR1)	Promote cancer progression, invasion and metastasis in breast cancer	Bind to NK1R, leading to cell death, the dying cells then release ssRNA that act on neighboring TLR7 to trigger pro-metastatic gene expression program		[124]

**Table 2:** Neurotransmitters and receptors and their effects on target cells and the tumor microenvironment

Neurotransmitters	Receptors	Effect on TME	Reference
Acetylcholine (ACh)	α7-nicotinic acetylcholine receptor	Inhibit TNF-α produced by macrophages in spleen, ultimately limit systemic inflammation	[28]
	-	reduced CD8 + T-cell infiltration in PDAC	[29]
5-HT	-	Decrease circulating angiostatin levels, thus stimulate tumor vascularity in colon tumors	[44]
Dopamine	D2	Lead to endocytosis of VEGF receptor 2, inhibit VPE/VEGF pathway, thus inhibit angiogenesis	[68]
		Promote tumor vessel normalization	[69]
Adrenalin and noradrenaline	α-adrenergic receptors and β-adrenergic receptor	Manipulate immune cells in TME to drive tumor, such as induce CD8^+^T cell exhaustion, suppress proliferation of CD8^+^T cell, increase infiltration of tumor-associated macrophages, thus promote progression and metastasis	[87–89]
		Stimulate vascularization	[96–98]
γ-Aminobutyric acid (GABA)	GABAAR	Induce macrophage infiltration in PDAC	[113]
	GABABR	Suppress CD8 + T-cell intratumoral infiltration	[114]
Neuropeptide Y (NPY)	NPY5 receptors	Promote myeloid cell infiltration and IL-6 release via STAT3 signaling pathway in prostate cancer	[126]

## Data Availability

No datasets were generated or analysed during the current study.

## Abbreviations

ACh: Acetylcholine
AChRs: ACh receptors
CHRM3: Cholinergic muscarinic type 3 receptor
NGF: Nerve growth factor
FAK: Focal adhesion kinase
YAP: Yes1 associated transcription regulator
CaM/CaMKK: Calmodulin/calmodulin-dependent protein kinase kinase
CHRM1: Cholinergic muscarinic type 1 receptor
CHRM4: Cholinergic muscarinic type 4 receptor
PCa: Prostate cancer
VGLUT: Vesicular glutamate transporter
GAKP: Guanylate kinase-associated protein
CRPC: Castration-resistant prostate cancer
HSPC: Hormone-sensitive prostate cancer
PDAC: Pancreatic ductal adenocarcinoma
CSC: Cancer stem cell
NET: Neutrophil extracellular trap
CNS: Central nervous system
mGluRs: Metabotropic glutamate receptors
iGluRs: Ionotropic glutamate receptors
AMPARs: α-amino-3-hydroxy-5-methyl-4-isoxazolepropionic acid receptors
NMDARs: N-methyl-D-aspartate receptors
PNETs: Pancreatic neuroendocrine tumors
FMRP: Fragile X mental retardation protein
HSF1: Heat shock transcription factor 1
B2BM: Breast-to-brain metastasis
PNS: Peripheral nervous system
TPH: Tryptophan hydroxylase
*5-HTP*: 5-hydroxytryptophan
AADC: Aromatic amino acid decarboxylase
CRC: Colorectal cancer
TPH2: Tryptophan hydroxylase 2
HTR: 5-HT receptors
MMP-12: Matrix metalloproteinase-12
HCC: Hepatocellular carcinoma
PDACs: Pancreatic ductal adenocarcinomas
GAPDH: Glyceraldehyde-3-phosphate dehydrogenase
NPY: Neuropeptide Y
CAF: Cancer-associated fibroblasts
H3Q5ser: Serotonylation of histone H3Q5
TGM2: Transglutaminase 2
DA: Dopamine
AC: Adenylyl cyclase
VPF: Vascular permeability factor
VEGF: Vascular endothelial growth factor
DR: Dopamine receptors
DRD1: D1 dopamine receptor
DRD2: D2 dopamine receptor
GBM: Glioblastoma
TH: Tyrosine hydroxylase
HIF-1α: Hypoxia-inducible factor-1α
VHL: Von Hippel-Lindau
DDC: Dopa decarboxylase
MAOA: Monoamine oxidase A
*β-ARs*: β-adrenergic receptors
NE: Norepinephrine
ICB: Immune checkpoint blockade
MDSCs: Myeloid-derived suppressor cells
DC: Dendritic cells
α2-AR: α2-adrenergic receptors
CREB: cAMP response element-binding protein
HDAC2: Histone deacetylase-2
TSP1: Thrombospondin-1
GABA: γ-Aminobutyric acid
GAD: Glutamate decarboxylase
GATs: GABA transporters
GABAAR: GABAA receptors
GABABR: GABAB receptors
GABACR: GABAC receptors
GPCR: G protein-coupled receptor
MAPK/ERK: Mitogen-activated protein kinase/extracellular signal-regulated kinase
NF-κB: Nuclear factor κB
ISCs: Intestinal stem cells
EMT: Epithelial-mesenchymal transition
VIP: Vasoactive intestinal polypeptide
SP: Substance P
TACR1: Tachykinin receptor 1
TLR7: Toll-like receptor 7
NETs: Neuroendocrine tumors
MTC: Medullary thyroid cancer
CGRP: Calcitonin gene–related peptide
cAMP: Cyclic Adenosine Monophosphate
KLF2: Kruppel Like Factor 2
NPY: Neuropeptide Y
